# Optimal configuration of on-scalp OPMs with fixed channel counts

**DOI:** 10.1162/IMAG.a.22

**Published:** 2025-05-30

**Authors:** Jan Mathijs Schoffelen, Teresa Cheung, Svenja Knappe, Robert Oostenveld

**Affiliations:** Donders Centre for Cognitive Neuroimaging, Donders Institute for Brain, Cognition and Behaviour, Radboud University, Nijmegen, The Netherlands; FieldLine Medical, Boulder, CO, United States; School of Engineering, Simon Fraser University, Burnaby, BC, Canada; Surrey Memorial Hospital, Fraser Health Authority, Surrey, BC, Canada; Paul M. Rady Department of Mechanical Engineering, University of Colorado Boulder, Boulder, CO, United States; FieldLine Industries, Boulder, CO, United States; NatMEG, Karolinska Institutet, Stockholm, Sweden

**Keywords:** optically pumped magnetometer, OPM, magnetoencephalography, MEG, software shielding, sensor array, noise cancellation

## Abstract

Recent technological developments have brought optically pumped magnetometers (OPMs) within reach of the larger neuroscientific community. The current state-of-the-art consists of whole-head systems that measure the magnetic field at >100 locations. OPM sensors can be constructed to measure the field in either 1, 2, or 3 orientations. Consequently, the number of channels can differ from the number of sensors. This allows for magnetoencephalography (MEG) system designs with multiple measurement orientations at fewer locations, many locations with fewer orientations, or, ideally, many locations with multiple orientations. Yet, due to budget constraints, starting OPM groups are typically getting fewer sensors than what could, in principle, be accommodated in a whole head helmet-like arrangement. Furthermore, implementing multiple orientations in a single sensor comes at a cost and hardware companies are still optimizing the trade-offs between sensor designs. To guide the OPM systems design, it is relevant to know the optimal spatial distribution and sensing orientation of OPMs. We performed a simulation study in which we kept the total number of channels constant. We compared 3 synthetic 192-channel OPM arrays that were composed of either monoaxial, biaxial or triaxial sensors, where the sensors were placed at either 192, 96, or 64 measurement locations, respectively. We simulated multiple instances of an MEG signal due to a dipolar source in the brain, contaminated by various combinations of noise, considering sensor noise, brain noise, and noise induced by head (and sensor) movements in the residual ambient magnetic field. An optimal design of the MEG system serves both to record the activity of the brain, as well as the environmental noise that is to be suppressed. We performed dipole fits and evaluated the localization error and the amplitude of the estimated dipole moment. We cleaned the data using various spatio(temporal) cleaning strategies prior to fitting the dipoles. Our observations confirm earlier work, in that 1) the sensing orientation radial to the head is in general more optimal to pick up activity from the brain than tangential directions, but that 2) adding sensing orientations tangential to the head surface helps in suppressing ambient noise sources. Yet, we did not observe a clear improvement comparing triaxial with biaxial OPMs. Given that triaxial sensing may come at the expense of reduced spatial sampling over the head and reduced signal-to-noise for individual channels, we conclude that, given a fixed number of channels, biaxial sensors may be preferred with the currently available technology.

## 1 Introduction

Neural activity in the brain produces electrical currents that can be measurable non-invasively on the scalp using electroencephalography (EEG) and around the head using magnetoencephalography (MEG). In contrast to EEG, where the measured signal is a scalar quantity, that is, the potential difference on the scalp surface, in MEG, the measured magnetic field around the head (i.e., the signal on a channel) reflects the scalar projection of the magnetic vector fields along the orientation of the sensor. Consequently, the MEG signal in each channel represents both a magnitude and an orientation. The development of MEG in general, and specifically, the advent of whole head superconducting quantum interference device (SQUID)-based MEG systems in the 1990s raised the question of optimal sensor configurations, not only discussing the optimal number and positioning of the sensors, but also the optimal sensing orientation. Based on early work (Ahonen et al., 1993;Vrba et al., 2004), commercial SQUID-based MEG systems have evolved to be equipped with around 300 channels (e.g., 248 for 4D/BTi, 275 for CTF, 306 for MEGIN/Neuromag).

Although some MEG systems have reference sensors placed far away from the head, to measure—in all directions—the magnetic fields due to external sources, the bulk of the sensors in commercial SQUID-based systems are close to the head and measure the field direction perpendicular to the head surface, the so-called normal, or radial component. This convention is based on ease of fabrication and the general perception that there were no convincing arguments for other configurations (although, seeHari & Ilmoniemi (1986)for an argumentation into the other direction, andVrba & Robinson (2002)highlighting advantageous situations for vector sensing). Since the early years of MEG, it has been argued that measuring the magnetic field components normal to the head surface is favored over the tangential ones, as the normal components are less affected by volume currents (Cuffin & Cohen, 1977;Hamalainen & Sarvas, 1989). Sensors normal to the head surface also facilitate the spatial interpretation of the signals as a topographic map and allow for forward/inverse modeling with simple volume conduction models. Although the magnetic field components tangential to the head surface are more strongly confounded by volume currents, they may still contain useful information that can be used to improve signal interpretation (Hochwald, 1997) or to separate the relevant signals from the noise (Brookes et al., 2021;Nurminen et al., 2013). Also, in specific experimental situations, where the exact location of superficial sources is known a priori, strategically placed tangential sensors may provide complementary information to radial sensing.

The advent of optically-pumped magnetometers (OPMs) for MEG (Johnson et al., 2010;Sander et al., 2012;Shah & Wakai, 2013;Xia et al., 2006) has rekindled the discussion on the optimal number of sensors and the optimal configurations (Brookes et al., 2021;Iivanainen et al., 2017;Marhl et al., 2022;Tierney et al., 2022). OPM-systems allow the end-user to more flexibly define sensor array geometries, and put sensors in arbitrary positions over the scalp, but also away from the head. Current OPM sensors often allow for the measurement of the magnetic field in two or three directions at the same location, thus providing directional information about the vector field, although the readout of the field in multiple directions may come at the expense of reduced signal-to-noise ratio (SNR) compared to a single direction. Other aspects of OPM-based systems that are different from SQUID-based systems, and thus relevant to consider, are the closer proximity to the scalp of the sensors and thereby an increased signal, larger intrinsic noise levels, the increased sensitivity to environmental noise due to the use of magnetometers rather than gradiometers, and additional movement-related noise if the subject is wearing the sensors and moving their head (with the attached sensors) in the ambient magnetic field.

The work presented here is concerned with the question of whether uniformly placed OPM sensors close to the head (with an overall fixed number of recorded signals) should measure the magnetic field in 1, 2, or 3 directions at each recording location. Previous work in relation to this question has mainly focused on a detailed comparison between monoaxial and triaxial arrays (Brookes et al., 2021;Tierney et al., 2022), and has argued in favor of triaxial arrays. Monoaxial arrays that measure the component of the magnetic field normal to the head surface are inherently most sensitive to signals that originate from the brain (Hughett & Miyauchi, 2000;Iivanainen et al., 2017). Sensor arrays which also contain channels that measure the magnetic field tangential to the head surface provide information about ambient noise that is most optimally spatially orthogonal to the information from brain sources. Thus, ambient noise may be filtered out more effectively, for instance using harmonic field correction (Tierney et al., 2022) or beamforming (Brookes et al., 2021). However, triaxial arrays ‘sacrifice’ two thirds of their channel count to measure the magnetic field in the tangential directions that are less informative with respect to signals coming from the brain. Biaxial arrays, on the other hand, will only have half of their channels oriented sub-optimally for brain activity. Here, we hypothesize that the tangential sensitivity of half rather than two-thirds of the channels may provide a sufficient account of the ambient noise fields. Biaxial sensor arrays, thus, may strike an optimal balance between signal and noise sensitivity, and therefore may be at least as good as triaxial OPM arrays with the same number of channels.

## 2 Methods

We performed a series of simulations to evaluate the localization accuracy of focal dipolar sources, for three configurations of 192-channel synthetic OPM sensor arrays. We simulated OPM channel data, combining dipolar sources with combinations of spatially white sensor noise, spatially colored background brain “noise”, and artifactual signals caused by movement of the head and sensor arrays in a static ambient magnetic field. Then, we quantified the localization error of a single dipole fitted to the data, after the application of four different offline noise suppression algorithms. The simulations were implemented in MATLAB (version 2023b), using FieldTrip (Oostenveld et al., 2011) and custom-written code. A schematic of the methods is shown inFigure 1.

**Fig. 1.**
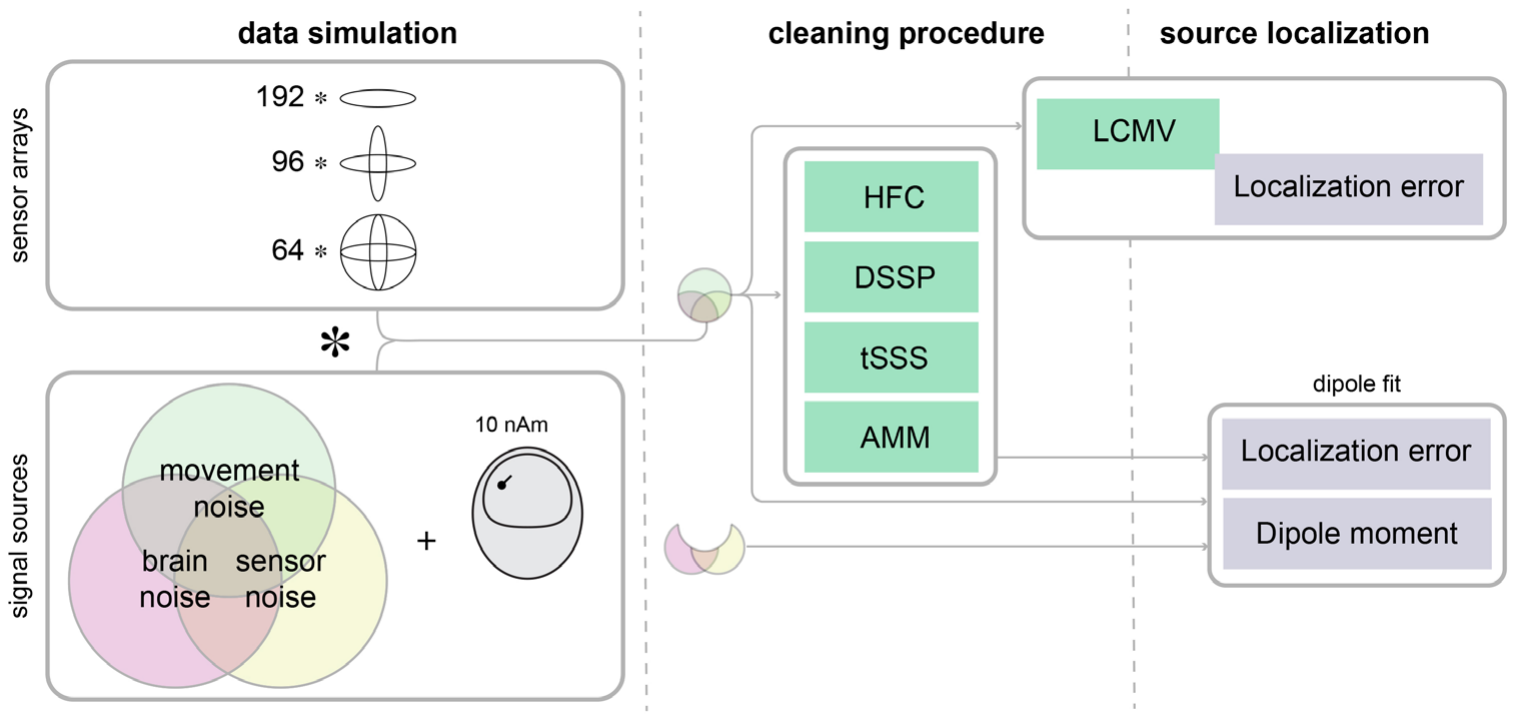
Schematic overview of the analysis approach.

### 2.1 Sensor array configurations

We compared synthetic sensor array configurations with a fixed number of channels, yet with different spatial distributions and orientations. Specifically, we compared arrays with either 192 monoaxial magnetometers, 96 biaxial magnetometers, or 64 triaxial magnetometers, each resulting in 192 channels. This number of channels was selected, because it is within the range of the current state-of-the art whole head OPM systems. The sensors were regularly distributed over the upper half of the head surface, which we modeled as an ellipsoid with cardinal axes lengths of 15 cm (x-axis: left-right), 18 cm (y-axis: posterior-anterior), and 16 cm (z-axis: inferior-superior). The origin of the coordinate system was the center of the ellipsoid.

**Fig. 2.**
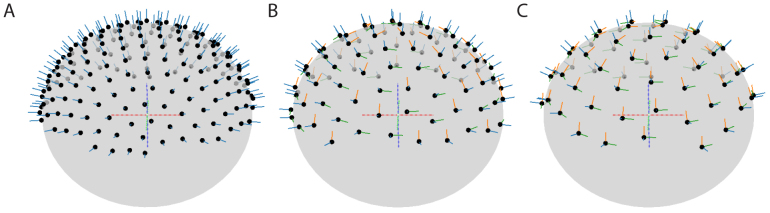
Spatial distributions and orientations of magnetometers in the three configurations of (A) 192 monoaxial magnetometers, (B) 96 biaxial magnetometers, and (C) 64 triaxial magnetometers.

The magnetometers of the monoaxial sensor array were oriented such that they were sensitive to the field perpendicular to the head surface, that is, with radial orientation. The biaxial sensor array consisted of pairs of magnetometers, one of which with a radial orientation, and the other with an orientation tangential to the head surface. Across sensor locations, the tangential orientation alternated between the azimuthal and polar orientation. The triaxial sensor array consisted of triplets of magnetometers, which sampled—at each of the 64 locations—the magnetic field in the radial and two perpendicular tangential directions.Figure 2shows an overview of the sensor arrays, where the channels are depicted as circles, indicating the plane perpendicular to their sensitivity direction.

### 2.2 Sensor array sensitivity analysis

Earlier work has shown that the orientation of magnetic sensors relative to the head surface affects their sensitivity to primary currents originating from the brain (Cuffin & Cohen, 1977;Marhl et al., 2022), where an orientation leading to the sensor picking up the field component normal to the head surface (radial orientation) is deemed optimal. We quantified this by exploring the values on the diagonal of the Gram matrix of a leadfield that was computed for a 192-channel triaxial synthetic sensor array (i.e., sampling the magnetic field at 64 distinct locations in 3 directions). The values on the diagonal of the Gram matrix reflect the amount of signal that a given sensor picks up from the sources. We modeled the sources as dipoles, distributed on a densely sampled regular 3-dimensional grid with a spacing of 2.5 mm within the brain compartment, which we modeled as the upper half of an ellipsoid with cardinal axes of 12.9, 15.4, and 13.7 cm. For each of the 46,783 locations in the brain compartment, we computed a forward model for a dipole in the x, y, and z direction, yielding a leadfield matrix with ~140,000 columns, each of which reflected the sensor topography of a dipole with unit amplitude. The Gram matrix was constructed as the inner product of this leadfield matrix, normalized with the total number of sources.

### 2.3 Dipole data simulation

To simulate signals due to active sources, we computed the forward solutions for dipoles placed at a regular 3-dimensional grid with a spacing of 10 mm, and with a minimum z-coordinate of 20 mm above the horizontal plane. At each of the 580 selected locations, three dipoles were simulated with an amplitude of 10 nAm and an orientation along the axes of a local coordinate system, where the orientation of the locally defined x’, y’, and z’ axes was rotated relative to the cardinal x, y, and z-axes. The amplitude of 10 nAm was selected because it is considered to be a realistic amplitude of dipolar sources in real data. Each dipole’s local coordinate system was defined such that the local z’-axis was the most silent, that is, the most radial component, and the local x’/y’-axes spanned the tangential plane. Next, for the two tangential orientations of each of the simulated dipoles, we obtained MEG signals by adding the forward solution to every second column of the simulated noise matrices. Thus, all odd-numbered columns in the final simulated data matrices could be considered as baseline observations, that is, sensor topographies of background noise, and the even-numbered columns could be considered as active observations, that is, sensor topographies of background noise + signal-of-interest (here simulated with an amplitude of 10 nAm). Each simulated data matrix, consisting of 6,000 ‘time’ points, was cut into 45 overlapping segments of 600 samples (80% overlap). Each of these segments was individually used as an input to dipole fitting (either with or without software cleaning) and beamformer procedures.

### 2.4 Noise simulations

Several sources of noise were considered: spatially uncorrelated sensor noise, spatially correlated brain noise, and noise caused by movement.

#### 2.4.1 Spatially uncorrelated sensor noise

Spatially uncorrelated sensor noise was created with random and uncorrelated time courses with a Gaussian distribution with a mean value of 0 and a standard deviation of 2.0 pT. The value of 2.0 pT amounts to a noise floor of about 200 fT rms/sqrt(Hz) if a bandwidth of 100 Hz is assumed.

#### 2.4.2 Spatially correlated brain noise

To simulate spatially-correlated brain noise, we placed dipoles at 3,229 locations of a 3-dimensional grid with a spacing of 6.3 mm within the brain compartment. On each of these locations, three dipolar sources in the x, y, and z-direction, respectively, were simulated with a random time course taken from a Gaussian distribution with a mean value of 0 and a standard deviation of 1 nAm.

#### 2.4.3 Noise caused by movement

To model noise caused by movement of the participant’s head and the head-fixed OPM sensor array in the ambient field, we first mapped the residual DC magnetic field in the magnetically shielded room (MSR) at the Donders Institute. The static ambient field was measured with a fluxgate magnetometer (SENSYS FGM3D/75). The specific MSR (Vacuumschmelze AK3b) at the Donders Institute is typical for many MEG facilities and was installed in 2002. It consists of two layers of mu-metal and one layer of aluminium. The MSR has six homogeneous field compensation coils on the inside along the walls, ceiling and floor, but these were switched off during the recording of the residual DC field. At the center of the MSR, that is, where the head of a participant is typically placed for measurements with the cryogenic MEG-system, the residual magnetic field was sampled on 3 vertical planes, with a spacing of 23 cm. Within each of the planes, the spacing was 5 cm in the horizontal direction, and 10 cm in the vertical direction. At each sampled location, the magnetic field was measured in three orthogonal directions. To obtain a sufficiently high spatial resolution required for the simulations, the ambient field measurement was interpolated to the moving OPM sensors’ positions (see below), using a b-spline interpolation as implemented in SPM and FieldTrip “ft_leadfield_interpolate”. On average, the absolute residual DC field amplitude was 50.1 nT, and the average first and second order gradients were 6.7 nT/m and 35.6 nT/m^2^, respectively.

We obtained head movements from a real MEG measurement with a representative young adult participant, and combined this with the ambient field measurement in the MSR. The head position was measured using the CTF-device at the Donders Institute, which samples the position of 3 head localization coils at a frequency of 10 Hz. The experimental MEG session resulted in about 10 minutes of movement data (~6,000 samples). The positions of the three coils at the nasion and left and right ear canals were converted into a series of affine transformation matrices reflecting the translation and rotation of the circumcenter of the localization coils relative to the start of the measurement. Starting from the OPM sensor array centered in the virtual box in which the ambient fields had been measured, we projected the ambient vector fields onto the translated and rotated OPMs, resulting in a time series of the OPM positions and orientations, as well as a time series of the ambient field picked up by the OPMs. In addition, we also simulated the effect of moving the sensor array in a uniform magnetic field. For this, we created a uniform ambient vector field by replacing the position-dependent measurement by the median across measurement locations, for the three orientations separately.

**Fig. 3.**
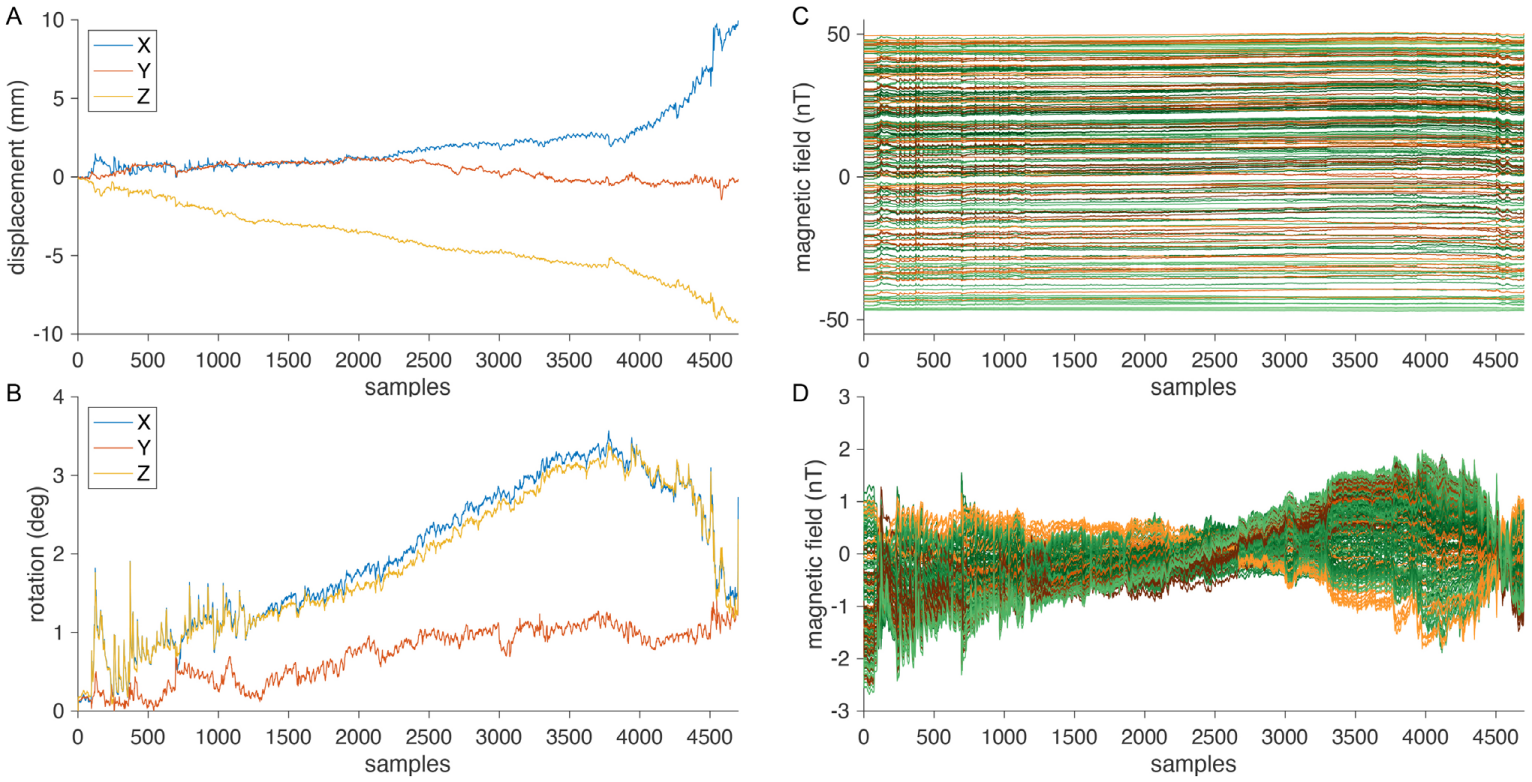
Movement time series used to generate the ambient noise measurements for the different sensor arrays. Displacement (A) and rotation angle (B) of the circumcenter of three head position coils during a realistic SQUID-MEG measurement. (C) Simulated ambient field as measured by a 192-channel triaxial array, at 64 locations (green lines: channels with tangential orientation, orange lines: channels with radial orientation). (D) Simulated ambient field as in (C), but mean subtracted.

For illustration purposes,Figures 3A and Bshow the head movement traces for translation and for rotation, respectively.Figures 3C and Dshow corresponding simulated MEG signals, using the measured and interpolated non-uniform residual field in the MSR, for a 192-sensor array with 64 triaxial channels, prior to and after subtraction of the per-channel mean field, respectively. Green-colored lines reflect channels with a tangential orientation, and brown/orange lines reflect channels with a radial orientation. Specifically, the rotation of the sensor array in the ambient magnetic field leads to substantial signal fluctuations of up to 4 nT. The standard deviation over time of the channels with radial orientation was 0.67 nT, similar to that of the channels with tangential orientations of 0.54 nT.

### 2.5 Data cleaning

An important consideration for the inclusion of the tangential directions in the OPM sensors is their ability to improve the algorithmic suppression of noise. Hence, we investigated six different cleaning procedures to mitigate the ambient field contamination: 1) simple baseline subtraction, 2) harmonic field correction (HFC) (Tierney et al., 2022), 3) dual signal subspace projection (DSSP) (Sekihara et al., 2016), 4) temporal signal space suppression (tSSS) (Taulu et al., 2005), 5) adaptive multipole modeling (AMM) (Tierney et al., 2024), and 6) linearly constrained minimum variance (LCMV) beamforming (Van Veen et al., 1997). The first technique operates channel-by-channel and does not use any spatial information. The latter five procedures are spatial filtering techniques, which use linear combinations of the measurement channels to remove unwanted features from the data. The algorithms differ in the details of the heuristics used to estimate the spatial filters.

*Baseline subtraction*was performed simply by subtracting the average signal in the baseline periods from the average signal in the active periods. Note that, if the spatiotemporal distribution of the ambient field in the baseline is the same as in the active period, a baseline subtraction would effectively clean the ambient noise. The*HFC*method is a spatial regression technique that removes low-order irregular spherical harmonic components from the data. The*DSSP*,*tSSS*, and*AMM*cleaning procedures consist of two steps, first a spatial—time invariant—linear projection, followed by a temporal linear projection, removing temporally correlated signals from the relevant subspace.*LCMV*beamforming is a spatial filtering technique that scans a pre-specified spatial volume for localized source activity, suppressing temporally uncorrelated noise, yielding a spatial map of (noise normalized) signal power, which allows for direct identification of the location with the maximum activity. With the exception of LCMV beamforming, the cleaning step was followed by a dipole fit using the FieldTrip routine “ft_dipolefitting” to estimate the location and moment parameters of the best fitting dipole. We evaluated HFC using spherical harmonics up to an order of 1 (denoted below as*HFC1*), and up to an order of 3 (denoted as*HFC3*). For the evaluation of tSSS cleaning, we used an order of 1 or 3 for the external compartment, denoted below as*tSSS1*and*tSSS3*, respectively. All procedures were implemented in MATLAB, using a core FieldTrip routine (DSSP: “ft_denoise_dssp”), or custom written code (HFC, tSSS, AMM, LCMV beamforming^*^).

The adaptive cleaning techniques (AMM, tSSS) were applied to each 600-sample data segment separately, and the covariance matrix, needed for the LCMV beamforming, was computed per segment as well.

### 2.6 Dipole fitting

After software cleaning, we computed average topographical data vectors for each segment, by subtracting the average of the odd-numbered baseline samples from the average of the even-numbered active samples. These topographical data vectors (45 instantiations per dipole location, per noise condition) served as input to the dipole fitting algorithm, using a standard non-linear dipole fit, with the simulated dipole location as a starting position for the non-linear search for the dipole model’s position parameters. Initial explorations showed that a computationally more demanding initial grid search for the identification of the starting position effectively did not yield better localization results overall.

## 3 Results

### 3.1 Sensitivity to signals from the brain depends on channel orientation

**Fig. 4.**
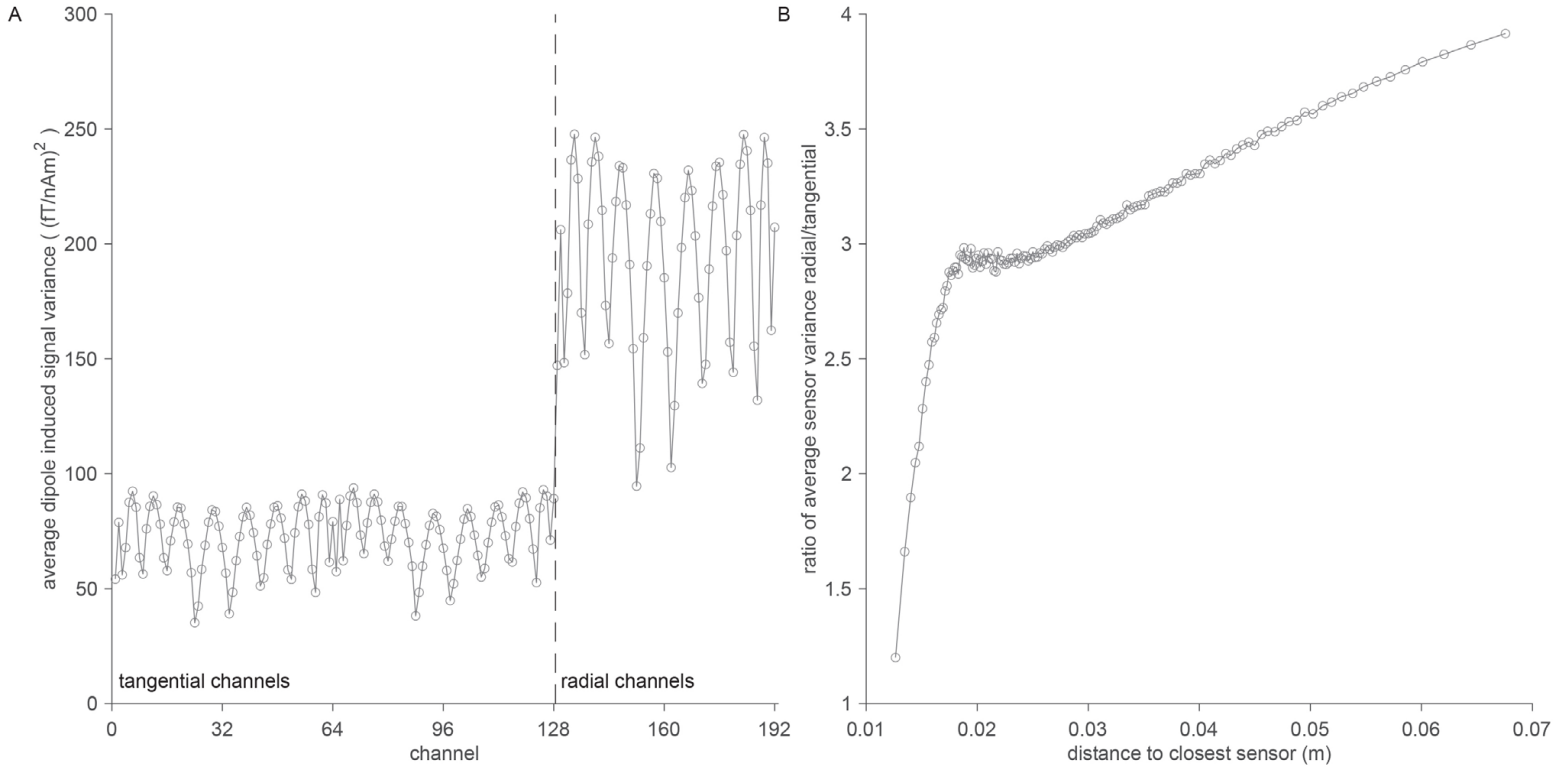
Sensor orientation affects sensitivity to signals from the brain. (A) Diagonal of Gram-matrix, reflecting the signal variance due to ~140,000 dipolar sources within the brain compartment. (B) Ratio of radial/tangential variance for dipoles as a function of distance to the closest sensor.

Figure 4Ashows the values on the diagonal of the Gram matrix for the triaxial sensor array with 192 channels at 64 locations. Channels with a radial orientation relative to the head surface pick up a larger signal from sources originating from the brain compartment, as compared to channels with a tangential orientation, on average 2.7 times as much. This ratio, however, depends on the distance of the source to the channels.Figure 4Bshows the ratio between the variance on the radial and tangential channels, where the ratio was calculated from Gram matrices repeatedly computed on subsets of 1,000 dipoles, sorted according to their distance to the closest channel. The tangential channels are less insensitive to dipoles that are very close to any of the channels, as compared to more distant, deeper dipoles. Overall, this investigation confirms that channels with an orientation perpendicular to the surface of the head have the largest signal for sources within the brain.

### 3.2 Difference in dipole localization accuracy depends on the type of noise

We explored the effect of different types of noise on the quality of dipole fits to the simulated signals of the different sensor arrays. The following figures show the distribution of the dipole localization errors (left panels) and dipole amplitude estimates (right panels) for the different channel arrays. The figures summarize the results of 52.200 dipole fits (for 580 initial dipole locations, 2 tangential orientations per location, 45 noise realizations).

### 3.3 Effect of spatially white sensor noise

**Fig. 5.**
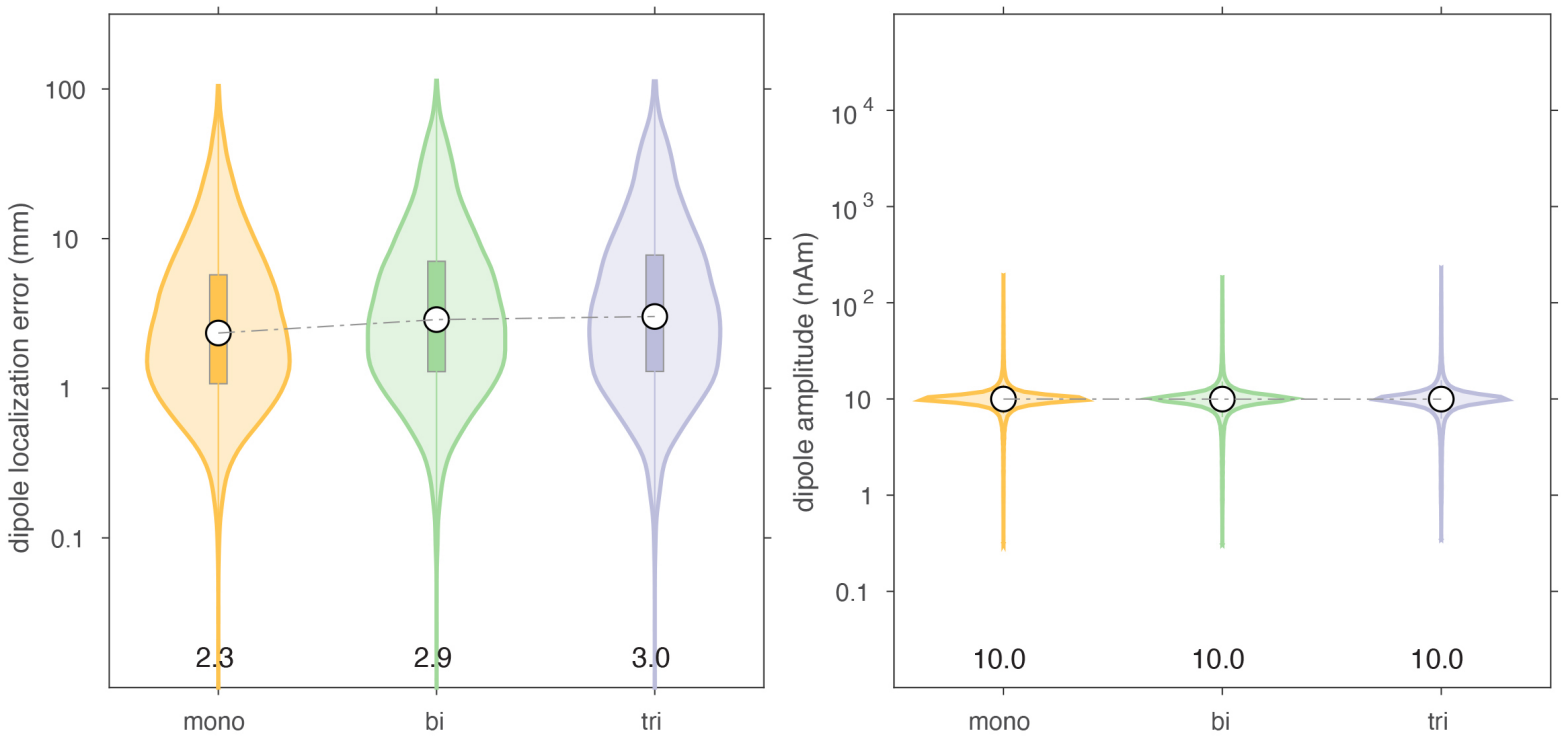
Comparison of dipole localization error and estimated dipole moment in the presence of spatially white sensor noise.

Figure 5shows the distribution of the dipole localization errors and reconstructed dipole amplitudes for simulations of the three different sensor arrays with added spatially white sensor noise. For the non-linearly estimated localization error, the monoaxial array performs better than the biaxial and triaxial arrays, where the median of the localization error is 2.3 mm for the monoaxial array, and 2.9 and 3.0 mm for the biaxial and triaxial arrays, respectively. We note that the marginal distributions are very similar across the three sensor arrays. The same observation holds for the reconstructed amplitudes, which are estimated linearly, and are centered very closely around the simulated amplitude of 10 nAm. Doubling the sensor noise in the simulations (using Gaussian noise with a standard deviation of 4 pT) increased the median localization error 4.9, 6.1, and 6.5 mm for the three sensor arrays (seeSupplementary Fig. S1).

### 3.4 Effect of spatially colored brain noise

**Fig. 6.**
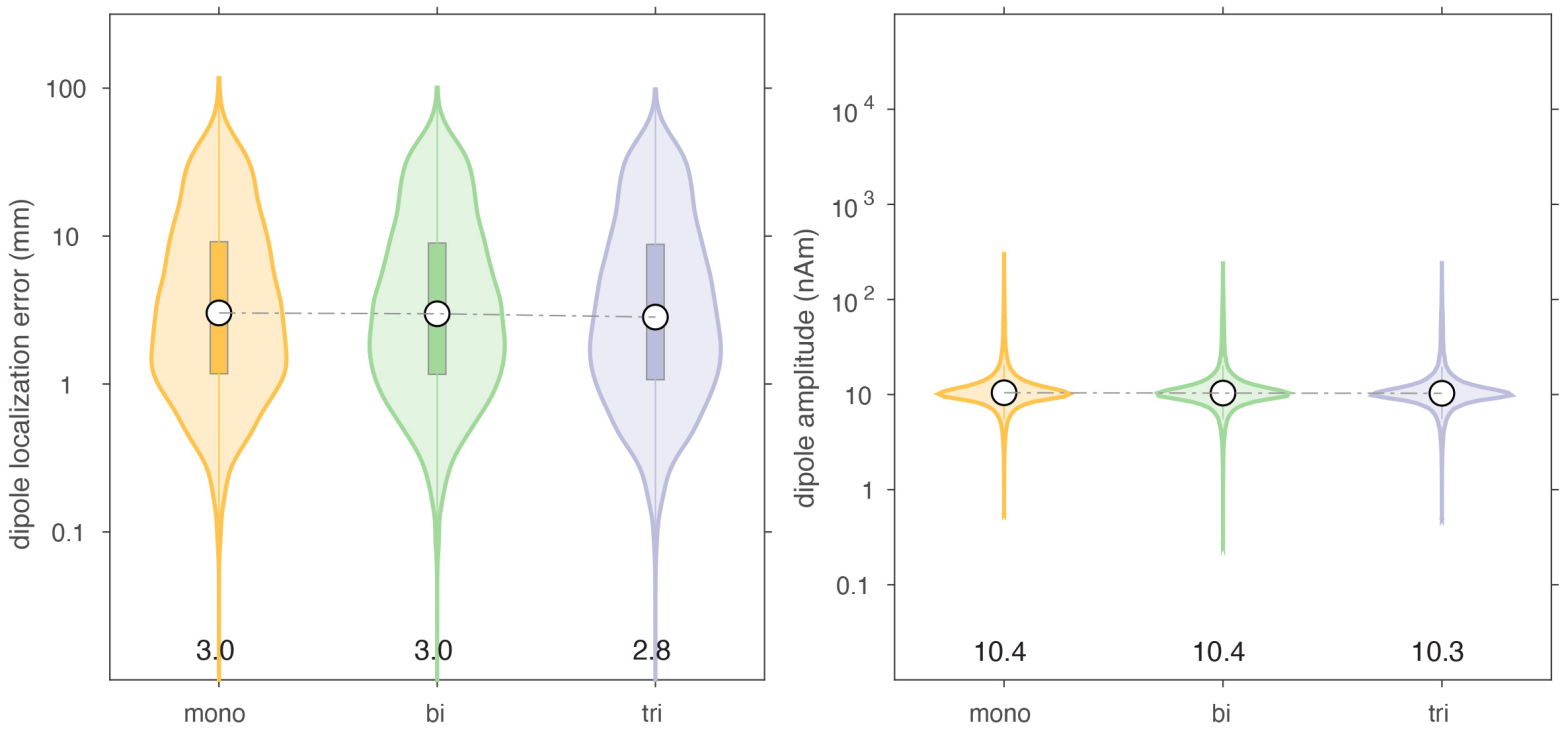
Comparison of dipole localization error and estimated dipole moment in the presence of spatially colored ‘brain’ noise.

The addition of spatially colored brain noise to the simulated data resulted in very comparable results for the different arrays, and did not indicate a clear advantage of one type of array over the other, at least not given the amount of added noise used in our simulations (Fig. 6).

### 3.5 Effect of movement noise

Movement noise was simulated by moving the sensor arrays through the residual background field. The background field had an amplitude that was at least 3 orders of magnitude larger than the signals coming from the brain compartment. This degraded the performance of the dipole fits substantially by inflating the localization error, as well as by reducing the reconstructed amplitude, as shown inFigure 7. In this context, the triaxial array clearly outperformed the mono- and biaxial arrays. For the triaxial array, the median localization error was 12.5 mm (IQR: [4.0–39.6]), and for the mono- and biaxial arrays this was 48.3 ([32.8–61.2]) and 35.2 mm ([6.7–60.2]), respectively. Here, the ‘baseline-correction’ of the sensor topographies, that is, the subtraction of an average of background signal from an average of background and dipole signal, was largely ineffective. When simulating the movement in a uniform background field, the localization performance was less bad for all three arrays (Fig. 8). Nevertheless, the triaxial array clearly outperformed the other array types, with a median localization error of 6.1 mm (IQR: [2.5–17.5]). Interestingly, in the absence of any other spatiotemporal cleaning besides baseline subtraction, the overall dipole localization accuracy of the triaxial array was in the range of what can typically be expected in realistic scenarios.

**Fig. 7.**
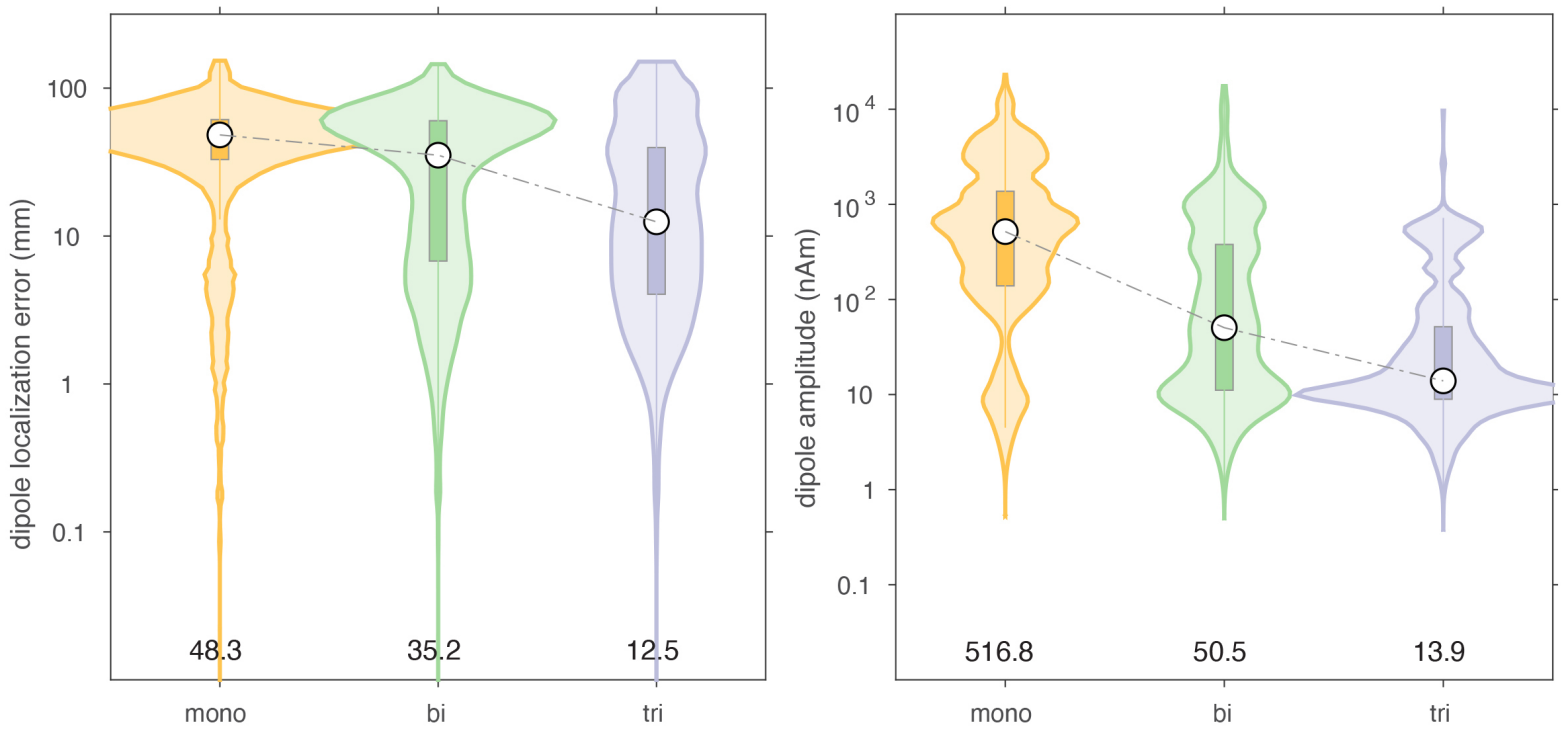
Comparison of dipole localization error and estimated dipole moment in the presence of a static non-uniform ambient field, through which the sensor array was moving.

**Fig. 8.**
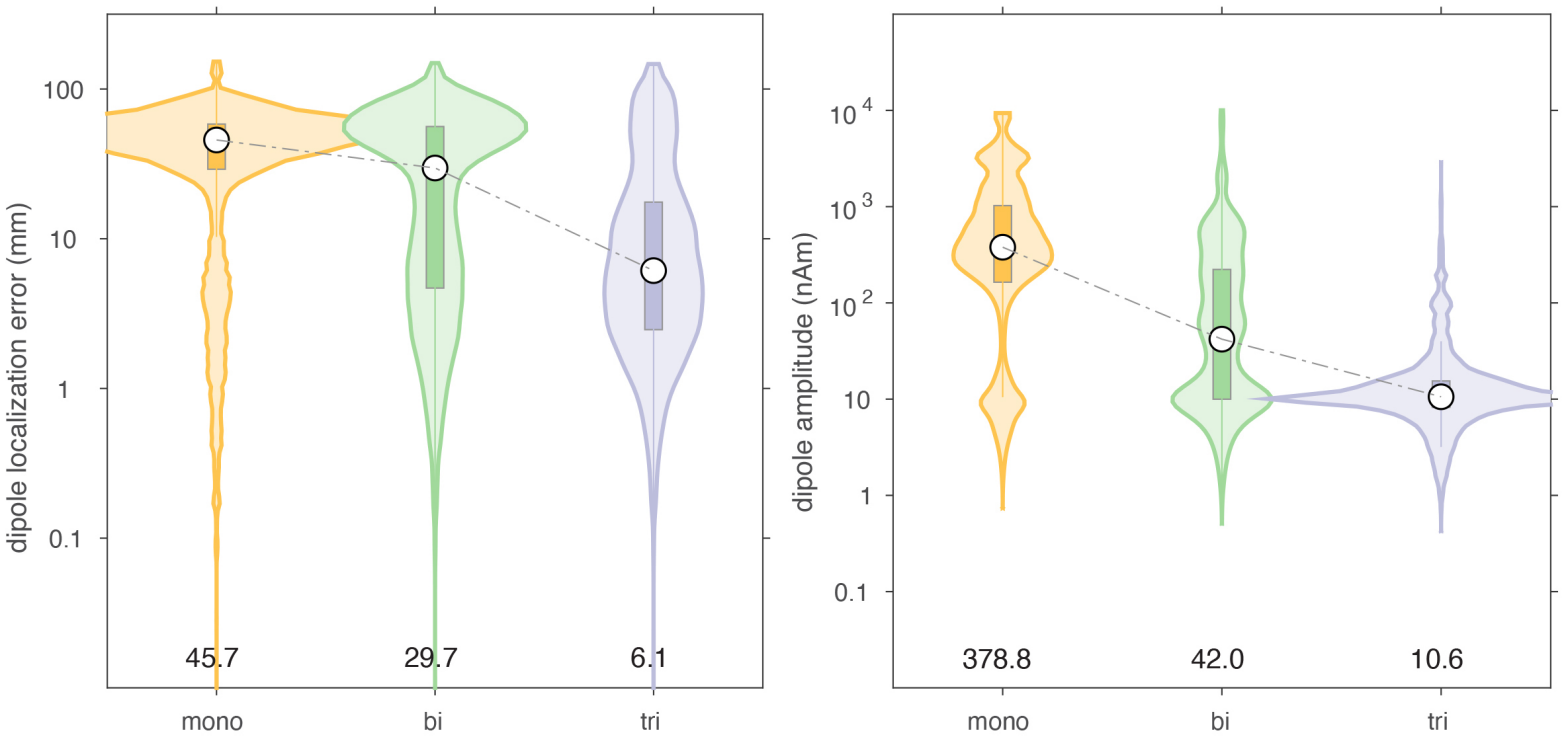
Comparison of dipole localization error and estimated dipole moment in the presence of a static uniform ambient field, through which the sensor array was moving.

### 3.6 Effect of signal cleaning on reconstruction behavior

**Fig. 9.**
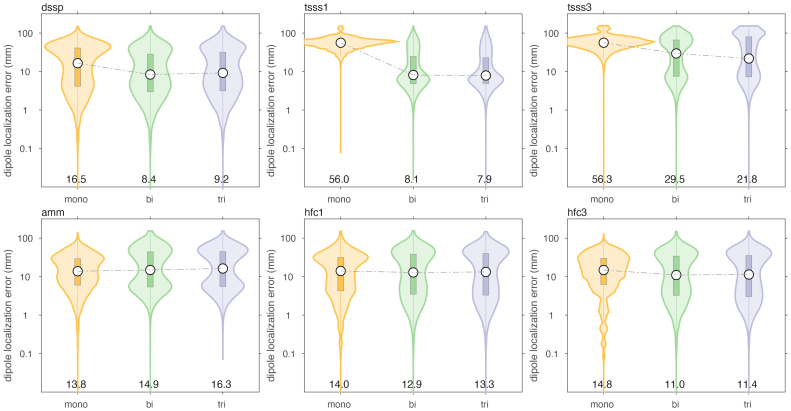
Comparison of dipole localization error of cleaned data, in the presence of sensor and brain noise, and a static non-uniform ambient field, through which the sensor array was moving.

We evaluated the effect of various spatial and spatio-temporal cleaning techniques on the dipole localization accuracy, typical for MEG data processing. It was not our intention to make an explicit comparison between the different cleaning methods, but rather to evaluate the dipole localization accuracy of the different arrays, independent of the specifics of the signal processing.Figures 9and10show the results when simulating movement-induced noise in a non-uniform background field, andFigures 11and12display the results from data simulated in a uniform background field.

**Fig. 10.**
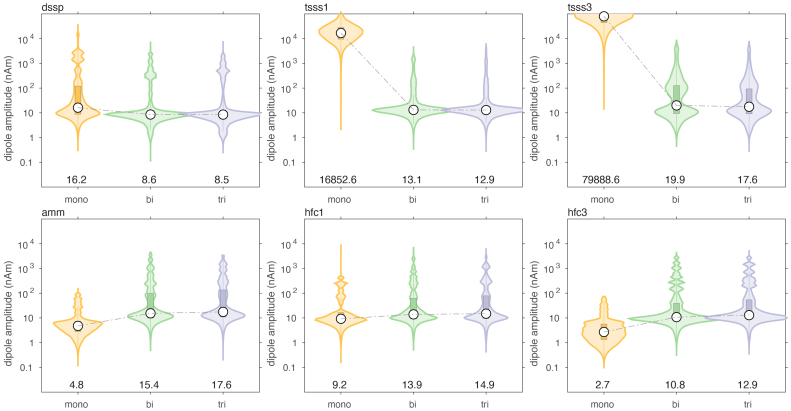
Comparison of the estimated dipole amplitude of cleaned data, in the presence of sensor and brain noise, and a static non-uniform ambient field, through which the sensor array was moving.

**Fig 11.**
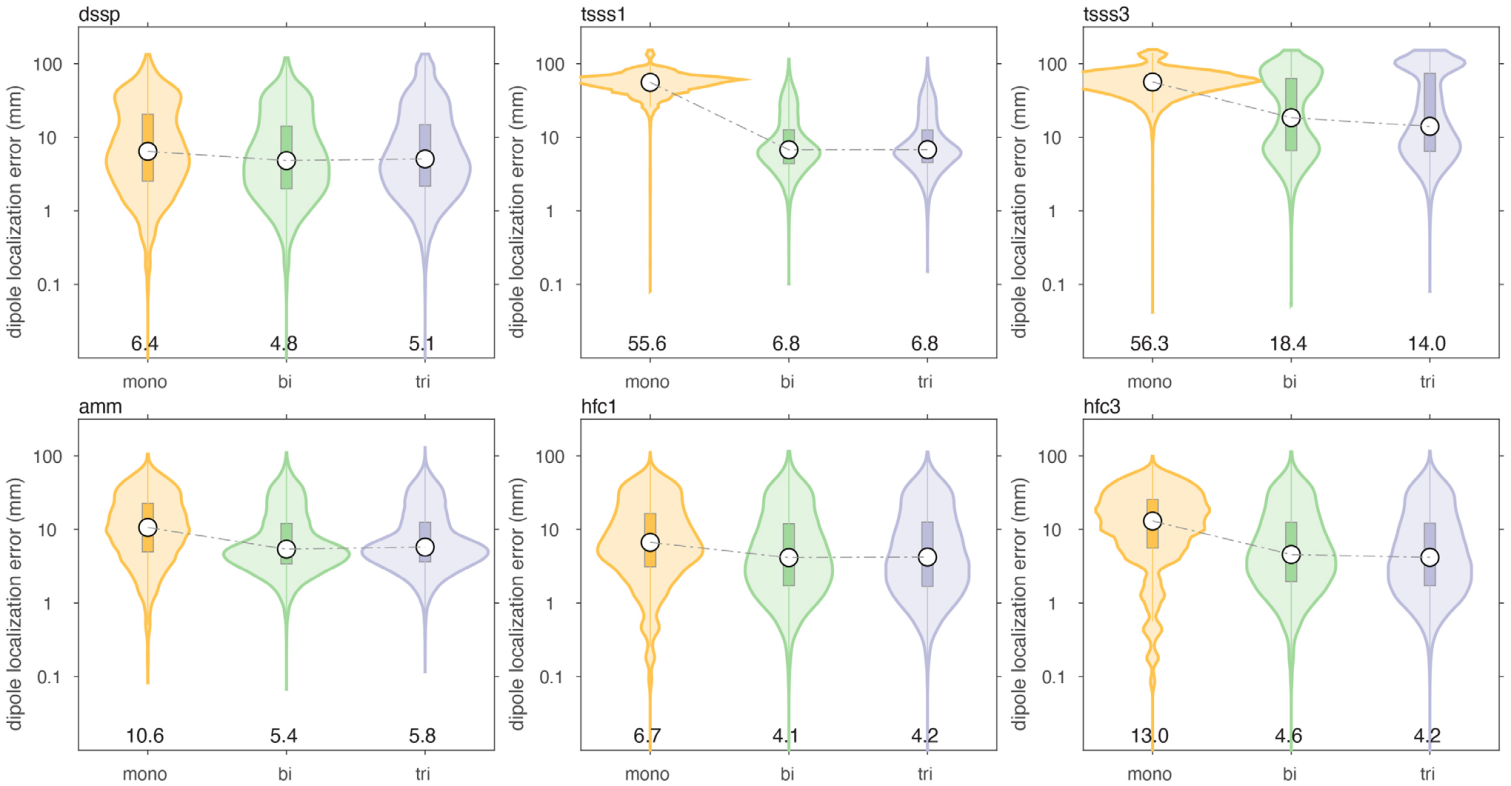
Comparison of dipole localization error of cleaned data, in the presence of sensor and brain noise, and a static uniform ambient field, through which the sensor array was moving.

**Fig. 12.**
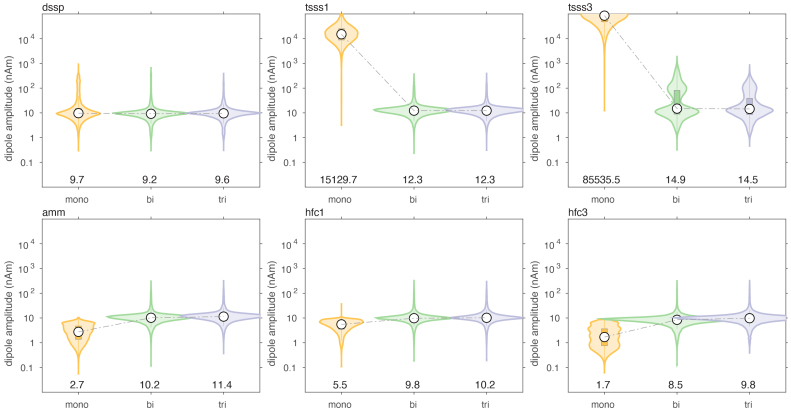
Comparison of the estimated dipole amplitude of cleaned data, in the presence of sensor and brain noise, and a static non-uniform ambient field, through which the sensor array was moving.

For both types of movement-induced noise, software cleaning overall greatly improved the dipole localization accuracy, compared to the situation where only baseline subtraction was performed. The largest improvement was observed for the mono- and biaxial arrays. For example, after DSSP cleaning, the median localization errors for the monoaxial, biaxial and triaxial arrays were 16.5 (IQR: [4.1–40.9]), 8.4 (IQR: [3.0–28.3]), and 9.2 (IQR: [3.1–31.6]) mm. The magnitudes of the reconstructed dipole moments were 16.2 (IQR: [8.8–120.4]), 8.6 (IQR: [6.1–12.5]), and 8.5 (IQR: [5.7–12.6]) nAm, respectively. In terms of localization error, the biaxial array performed slightly better than the triaxial array. In terms of dipole moment, the biaxial array performed very similarly to the triaxial array. It should be noted that the DSSP cleaning resulted in the median of the dipole moment being slightly underestimated with the biaxial and triaxial arrays, and overestimated with the monoaxial array. Finally, DSSP cleaning of data simulated with movement-induced noise in a uniform field resulted in an improved localization, compared to movement in a non-uniform field, with most notably the median localization error for the monoaxial array now also being below 10 mm (6.4 [2.5–20.5], 4.8 [2.0–14.1], 5.0 [2.2–14.8] for the 3 types of arrays).

The overall performance improvement in localization performance of the other cleaning techniques was qualitatively and generally comparable to the improvement observed with DSSP, although the results varied in the details. ComparingFigures 7and8withFigures 9and10, there was a mostly overall reduction in the localization error, and a more accurate reconstruction of the dipole amplitude. Also, the contamination of the signals by movement in a uniform field was generally better dealt with than the contamination of the signals by movement in a non-uniform field, that is, a field with spatial gradients.

Tables 1and2summarize the median localization error and the interquartile ranges (IQR), that is, the 25’th–75’th percentile, of the dipole moment magnitude for the various cleaning techniques, for the case of a non-uniform background field. In each column of the table, we highlighted in “underlined-bold-italic” and “italic” the sensor array for which the performance was deemed ‘best’. For the localization error this pertained to the lowest error, and for the IQR this pertained to the situation with the narrowest IQR that also included the simulated dipole amplitude. With the exception of the two types of tSSS applied to the monoaxial array data, and AMM/tSSS3 applied to the triaxial data, the cleaning resulted in a reduction of the localization error, and by consequence to a narrowing of the reconstructed amplitude’s IQR. Furthermore, for the monoaxial array, AMM and HFC3 resulted in an amplitude IQR with the 75’th percentile being below the simulated amplitude of 10 nAm. This suggests that those cleaning techniques may have a tendency to overcompensate (i.e., also remove part of the signal of interest) in monoaxial array data. Notably, after cleaning the localization quality for the triaxial array was not better than for the biaxial array. More specifically, out of the 7 cleaning methods for which the localization error could be compared, 3 methods yielded the lowest error for biaxial arrays, versus 2 methods for the triaxial arrays. For AMM the monoaxial array yielded the lowest error, and for LCMV the biaxial and triaxial arrays were at par. For the amplitude IQR, the biaxial array performed best with 3 cleaning methods (out of 6), while the triaxial array performed best in 2 cleaning situations.

**Table 1.** Median dipole localization error after various cleaning techniques, for the three different sensor arrays.

	*static, sensor noise only*	*movement*							
*median localization error (mm)*	*baseline subtraction*	*baseline subtraction*	*DSSP*	*AMM*	*tSSS1*	*tSSS3*	*HFC1*	*HFC3*	*LCMV*
*monoaxial*	***2.3***	*48.7*	*16.5*	***13.8***	*56.0*	*56.3*	*14*	*14.8*	*15.1*
*biaxial*	*2.9*	*34.2*	***8.4***	*14.9*	*8.1*	*29.5*	***12.9***	***11.0***	***8.7***
*triaxial*	*3.0*	***15.1***	*9.2*	*16.3*	***7.9***	***21.8***	*13.3*	*11.4*	***8.7***

**Table 2.** IQR (25’th–75’th percentile) of dipole moment magnitude after various cleaning techniques for the three different sensor arrays.

	*static, sensor noise only*	*movement*						
*IQR (25% 75%) (nAm)*	*baseline subtraction*	*baseline subtraction*	*DSSP*	*AMM*	*tSSS1*	*tSSS3*	*HFC1*	*HFC3*
*monoaxial*	***9.1*** ***10.9***	*144* *1360*	*8.8* *120*	*2.8* *7.2*	*9589* *28152*	*44889* *131191*	***6.5*** ***16.3***	*1.3* *5.6*
*biaxial*	*8.9* *11.0*	*11.3* *347.3*	***6.1*** ***12.5***	***9.9*** ***98***	*9.8* *19.2*	*9.4* *126.4*	*9.2* *62*	***7.9*** ***38.8***
*triaxial*	*8.8* *11.1*	***8.8*** ***49.5***	*5.7* *12.6*	*11* *135*	***9.7*** ***17.6***	***9.2*** ***92.8***	*9.5* *78.5*	*9.2* *53.8*

## 4 Discussion

In this paper, we performed simulations of OPM data to compare the performance of different sensor-array designs to localize and estimate the amplitude of dipolar sources embedded in various types of noise. Specifically, we compared sensor arrays with the same total number of channels but with different distributions of their sensitive axes. We find that monoaxial arrays with radial sensitivity perform best in situations with sensor noise only and without movement-induced noise. In the presented simulations, median localization error for a radial monoaxial array was around 2.3 mm, versus 2.9–3.0 mm in the biaxial and triaxial arrays. These errors are, in fact, quite low overall, and are likely the consequence of the fact that each dipole fit was performed on a field distribution constructed from a baseline corrected average of 300 samples (see Methods). When we double the sensor noise in the simulations from 2 to 4 pT (Supplementary Fig. S1), the median localization error increased to 4.9, 6.1, and 6.5 mm for the three sensor arrays, thus indicating a consistent deterioration of localization accuracy of about 25% (biaxial) to 30% (triaxial) relative to the monoaxial sensor array. These observations are in line with earlier work (Iivanainen et al., 2017), estimating that radial channels offer roughly 40% more average information capacity per channel than tangential ones. Thus, orienting more channels radially appears beneficial in order to generically optimize the neural source detection capability of the sensor array, consistent with designs of current SQUID-based MEG systems.

When movement of the OPM sensor arrays along with the movement of the head is considered, the non-linear rotational component of the sensor movement in the background field greatly impacts the results, causing simple linear baseline subtraction to ‘fail’, and additional cleaning methods are advisable. Without additional cleaning, the triaxial array performed best. After the application of various state-of-the-art cleaning algorithms, based on spatial filtering, we observed a variable improvement of the localization accuracy, where the arrays with tangentially placed sensors overall outperformed the monoaxial array. This observation is in line with earlier work, which argued that the addition of tangential channels in a sensor array improves the spatial separability of brain sources from ambient noise sources (Brookes et al., 2021;Nurminen et al., 2013;Tierney et al., 2022). Interestingly, according to Brookes et al. (Brookes et al., 2021), where they used beamformers for noise rejection, significant improvements can already be achieved with 5 transversely oriented OPM channels, in a monoaxial array with otherwise radially oriented channels. Thus, to leverage the reduced spatial correlation between ambient and neural field patterns, it may not be necessary to ‘sacrifice’ two-thirds of the channels to be less sensitive to neural sources. Indeed, we did not observe a significant difference in the performance of biaxial and triaxial arrays when comparing various standard implementations of cleaning strategies. With some care we might even conclude that the biaxial arrays performed slightly better than triaxial arrays, given that—in a direct comparison across the three types of arrays, and in the scope of the cleaning methods used—the biaxial outperformed the triaxial array more often than the other way around.

It was not our intention to provide a detailed examination of the performance of the different cleaning methods. We are aware that specific implementational details and parameter optimizations could affect their relative performance in individual situations. For instance, the efficacy of the SSS technique to separate sources originating from the inside and the outside of the modeled sphere may be already somewhat limited in the monoaxial arrays. Also, techniques such as HFC and AMM are designed mathematically to provide similar interference reduction across arrays. HFC and AMM cleaning may be tweaked to better retain the inside signal, which may lead to a reduction in the underestimation of the dipole moment, for example, by incorporating the spatial projections in the forward model used for dipole fitting. Despite this, some generic observations can be made: With the exception of tSSS applied to monoaxial arrays, all cleaning methods are effective in bringing down the median dipole localization error to about 1.5 cm—and sometimes substantially lower—in data contaminated with noise due to movements in a non-uniform background field. If the background was uniform, that is, without any spatial gradients, localization errors were even lower (median between 4 and 7 mm for the biaxial and triaxial arrays, with the exception of tSSS3). Future work is needed to more systematically evaluate how and whether specific array configurations and cleaning methods interact.

In our simulations, we chose to use point dipoles as a source model. Notably, the simulated dipoles were placed on a 3-dimensional grid covering the whole brain and encompassing both deep and superficial locations. In future work, it will be relevant to investigate in more detail the effect of source parameters and more complex source configurations, for instance dipole depth, source extent, or number of and distance between sources.

It could be argued that additional channels at the same sensor location come at negligible cost. In other words, why would one use biaxial sensors in the first place, if one could just as easily obtain magnetic field measurements in three orientations at each sensor location? So far, continuous high-fidelity extraction—with a single-beam zero-field magnetometer—of all three vector components has not yet been demonstrated. Two axes can easily be measured with low noise, but the third axis will exhibit a higher noise floor because it is less efficient to measure the spin vector in the direction of the spin-orientation. In order to extract all three axes with a low noise floor, two biaxial sensors can be integrated into the same housing. This does not only require more (or more complicated) components but may also result in a higher standoff of at least one axis. Furthermore, measuring additional axes simultaneously increases the fundamental noise floor by sqrt(2) or sqrt(3) for biaxial and triaxial measurements, respectively. Also, controlling cross-axis projection errors (CAPE) (Borna et al., 2022) in multi-axis sensors, causing a distortion of the measured field, is a much more complex problem than in single-axis sensors and an ongoing area of development. Incorporation of a realistic extra penalty on the sensor noise with multi-axis sensing is a relevant avenue for future simulation studies. We have not considered spatially-correlated changes to the gain or orientation of the sensors due to common interference or movement in the assumption that closed-loop operation in three axes will make this effect negligible.

Another ‘cost’ to be considered is the OPM sensor lifetime. Most commercial alkali sensors use very low-power lasers, so called vertical-cavity surface-emitting lasers (VCSELs). Splitting the laser beam while maintaining the same intensity along each laser path requires higher laser power, which will typically lead to a reduced lifetime of the laser. Finally, triaxial measurements require higher heat load if the sensitive volume for laser beam is kept constant, which increases the surface temperature of the sensors, and may decrease subject comfort or require the OPM sensors to be placed further away from the head surface. We, therefore, argue that the cost of additional axes is not negligible but should be carefully considered.

## 5 Conclusions and Future Directions

We conclude that in situations of large transient external noise, for example when a subject is moving in a static ambient residual field,*non-radial sensing*improves the ability to separate noise from brain sources of interest. There does not seem to be a substantial benefit if the two components of the tangential field are sampled at the same location or at a neighboring sensor location, if the user incorporates spatial filtering into their analysis pipeline. Given that there are currently no ‘true’ triaxial zero-field sensors commercially available that measure the 3 axes with high fidelity in exactly the same location or volume (so not two volumes), at the same time (not interleaved) and with the same noise for the three orientations, the current solutions of biaxial sensors may be sufficient. Taking these considerations into account, we believe that a configuration where half the channels are oriented tangentially and half are oriented radially is a pragmatic approach to balancing neuronal sampling, interference mitigation and inherent sensor noise.

The biaxial sensor arrays used in our simulations were constructed to sample the azimuthal and polar tangential field components in an alternating fashion. This decision was based on our intuition that such a setup, in combination with the radial channels, provides a good 3-dimensional sensitivity for the ambient magnetic fields, independent of the relative position and orientation of the sensor array. We did not specifically consider whether this orientation scheme can be further optimized. This could be a topic for future research.

Also, in this paper, we only evaluated OPM-arrays with on-scalp magnetometer sensors. Other noise reduction techniques, such as gradiometry, or the use of external reference sensors may also be useful for addressing the ambient contamination of the signals. More work is needed to investigate in which way those techniques—which have proven their utility in the context of SQUID-based MEG—can be used in optically-pumped magnetometry as well.

## Supplementary Material

Supplementary Material

## Data Availability

Code and data needed to perform the simulations are available at:https://github.com/schoffelen/opm_simulations

## Footnotes

* HFC, tSSS, AMM, and LCMV beamforming are also available as core FieldTrip routines, but for computational efficiency we used custom code.
