# Supplementary material for: Optimal configuration of on-scalp OPMs with fixed channel counts
Source: Imaging Neurosci (Camb). 2025 May 30;3:IMAG.a.22. doi: 10.1162/IMAG.a.22 (PMC12320008; doi:10.1162/IMAG.a.22)

# Supplementary material

## Supplementary figure 1

*Distribution of dipole localization error and estimated dipole moment in the presence of white sensor noise with an amplitude of 4 pT.*

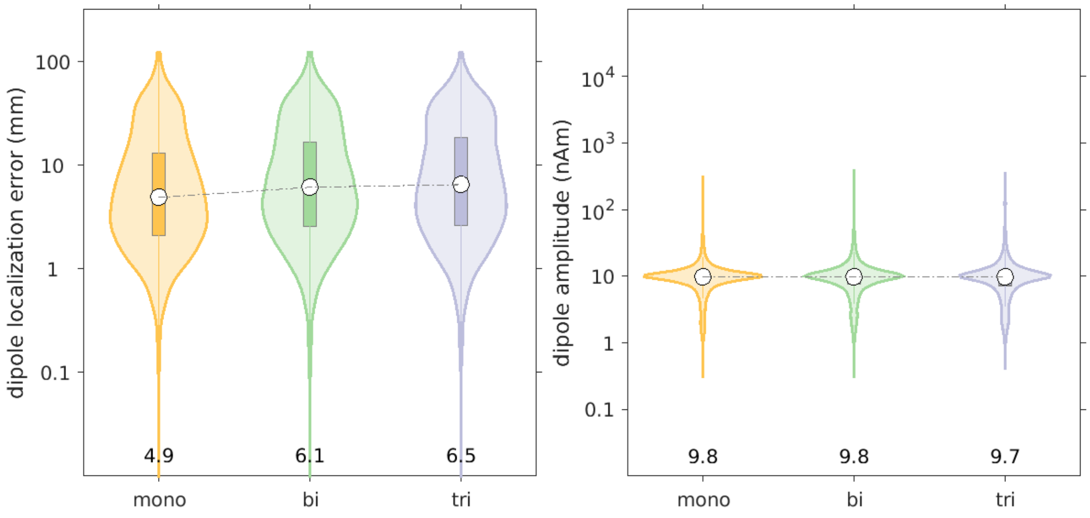

Supplement: Supplementary Material [file imag.a.22_supp.pdf]
